# Helios Expression Is a Marker of T Cell Activation and Proliferation

**DOI:** 10.1371/journal.pone.0024226

**Published:** 2011-08-30

**Authors:** Tatiana Akimova, Ulf H. Beier, Liqing Wang, Matthew H. Levine, Wayne W. Hancock

**Affiliations:** 1 Division of Transplant Immunology, Department of Pathology and Laboratory Medicine, The Children’s Hospital of Philadelphia and University of Pennsylvania School of Medicine, Philadelphia, Pennsylvania, United States of America; 2 Division of Nephrology, Department of Pediatrics, The Children’s Hospital of Philadelphia and University of Pennsylvania School of Medicine, Philadelphia, Pennsylvania, United States of America; 3 Department of Surgery, Penn Transplant Institute, Hospital of the University of Pennsylvania and University of Pennsylvania School of Medicine, Philadelphia, Pennsylvania, United States of America; Leeds University, United Kingdom

## Abstract

Foxp3+ T-regulatory cells (Tregs) normally serve to attenuate immune responses and are key to maintenance of immune homeostasis. Over the past decade, Treg cells have become a major focus of research for many groups, and various functional subsets have been characterized. Recently, the Ikaros family member, Helios, was reported as a marker to discriminate naturally occurring, thymic-derived Tregs from those peripherally induced from naïve CD4+ T cells. We investigated Helios expression in murine and human T cells under resting or activating conditions, using well-characterized molecules of naïve/effector/memory phenotypes, as well as a set of Treg-associated markers. We found that Helios-negative T cells are enriched for naïve T cell phenotypes and vice versa. Moreover, Helios can be induced during T cell activation and proliferation, but regresses in the same cells under resting conditions. We demonstrated comparable findings using human and murine CD4+Foxp3+ Tregs, as well as in CD4+ and CD8+ T cells. Since Helios expression is associated with T cell activation and cellular division, regardless of the cell subset involved, it does not appear suitable as a marker to distinguish natural and induced Treg cells.

## Introduction

T-regulatory cells (Tregs) constitute a functionally important subset of lymphocytes capable of fine tuning the immune response against pathogens and environmental stimuli [1]. Tregs are pivotal to maintaining self-tolerance and preventing autoimmunity, but are also involved in limiting physiologic immune and antitumor activity [2]. The ability to control Treg function could have major therapeutic potential for conditions ranging from autoimmune diseases and transplantation to malignancies, such that many investigators have begun to characterize Treg phenotypes and aspects of their biology. The transcription factor, Foxp3, is well recognized as central to Treg function [3], [4]. However, even Foxp3 is not absolutely Treg-specific, given its expression by activated T cells [5], and at least one report, some non-lymphoid cells [6], thereby limiting its utility as a universal Treg marker. Defining functional subsets of Treg is even more complicated, though Tregs can be divided into the two broad categories of natural occurring thymus-derived Tregs (nTreg), and peripherally induced Tregs (iTreg) that can develop from naïve T cells under a variety of conditions [7]. Both subsets share similar molecular signatures, including expression of Foxp3, high expression of CD25 [8] and CTLA-4 [9], and low expression of CD127 [10], and share multiple suppressive mechanisms [11]. These close similarities make ready discrimination of nTregs and iTregs nearly impossible. However, being able to determine the origin of a given Treg may be of importance in basic studies of Treg biology, or when monitoring the success of therapeutic interventions aimed at altering Treg production or function.

Some additional molecules have been proposed to discriminate between nTreg and iTreg. For example, CD31 (PECAM-1) is reportedly expressed by recent thymic emigrant CD4+ T cells but not by peripherally expanded naïve T cells [12], [13]. CD31 is cleaved and shed from the surface of human T cells upon activation via the T cell receptor (TCR) [14], and declines with aging, along with thymic involution [15]. Recently, several microarray studies indicated an up-regulation of the Ikaros family transcription factor, Helios, in Tregs [16], [17], [18]. Ikaros DNA-binding proteins are characterized by two highly conserved zinc finger domains that mediate DNA (N-terminal domain) and protein binding (C-terminal domain) [19]. The Ikaros family is comprised of 5 members; Pegasus and Eos are present in all tissues, whereas Ikaros, Aeolus, and Helios are selectively expressed in lymphocytes [20]. Recently, Thornton et al. reported that nTregs but not iTregs express Helios [21], thereby generating much interest in Helios and Treg subsets [22]. Considering the importance of identifying nTreg from iTreg, we decided to investigate the role of Helios in mice and human T cells using well-characterized molecules of naïve/effector/memory phenotypes, as well as Treg-associated markers.

## Results

### Helios co-expression with T cell and Treg-associated markers

**Figure 1:**
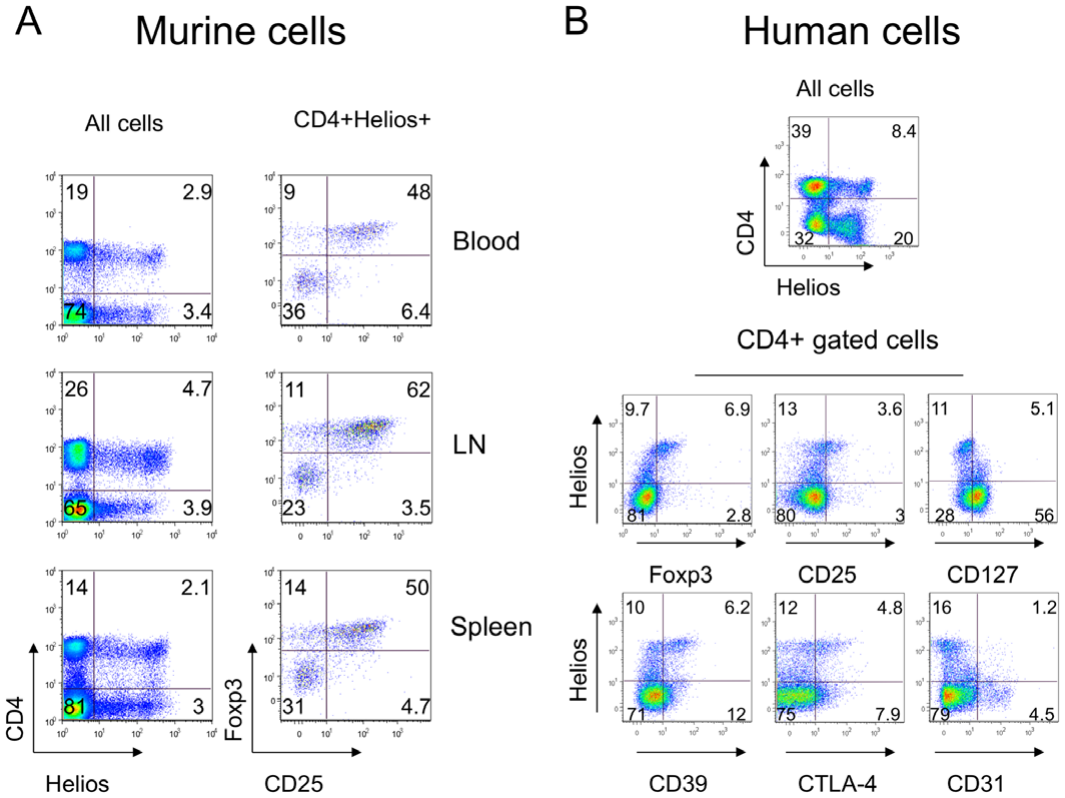
Helios expression was not restricted to CD4 cells, but among CD4+ cells correlated with Treg-associated markers. (A) Expression of Helios in murine lymphocytes (left column) and CD25+ Foxp3+ co-expression in CD4+ Helios+ gated subsets (right column) of blood (top), lymph nodes (middle) and spleen (bottom). (B) Expression of Helios in human lymphocytes (top) and co-expression of Helios with Treg-associated markers in cells gated for CD4 expression. Right column, bottom row shows absence of correlation between Helios and CD31, a marker of recent thymic emigrants cells. Data are representative of 5 experiments.

We first assessed Helios expression by flow cytometric analysis of human and murine peripheral blood mononuclear cells (PBMC), plus cells from murine lymph nodes and spleens. CD4+, CD8+ and CD4-CD8- cells expressed Helios, with CD4+ Foxp3+ Treg showed the highest Helios expression in both species (**Table 1**). There were no gender-based differences in Helios expression when tested using age-matched samples (data not shown). In mice, Helios+ T cells from lymph nodes and spleen were more likely to co-express Foxp3 and CD25 than PBMC (**Figure 1A**). In human CD4+ cells, the highest levels of Helios expression were associated with Foxp3, CD25, CD39, CTLA-4 (CD152) and low levels of CD127, while intermediately positive Helios+ cells included non-Treg cells (**Figure 1B**). Importantly, CD4+ Helios+ and CD4+ Helios- cells expressed CD31, a marker of recent thymic emigrant cells, almost equally (**Figure 1B**). Together, these data suggest that Helios might not be a specific marker of nTreg cells.

**Table 1:** Helios expression in mononuclear cells.

Cell population	% Helios+ murine cells (mean ± SEM)			% Helios + human cells (mean ± SEM)
	Blood	LN	Spleen	Blood
CD4+cells	13.4±0.8	12.3±1.2	11.2±0.6	12.6±1.2
CD4+ Foxp3+ cells	79.7±0.7	74.8±3.5	76.7±3	63.4±1.2
CD4+ Foxp3- cells	6.6±2	2.8±0.6	3.8±1.3	3.5±0.7
CD8+ cells	6.9±2.9	4.6±0.5	7.4±0.9	21.8±3.5
CD4- CD8- cells	3.8±0.3	4.4±1.2	3.2±0.4	16.1±2.8

### Helios expression and T-cell maturation

**Figure 2:**
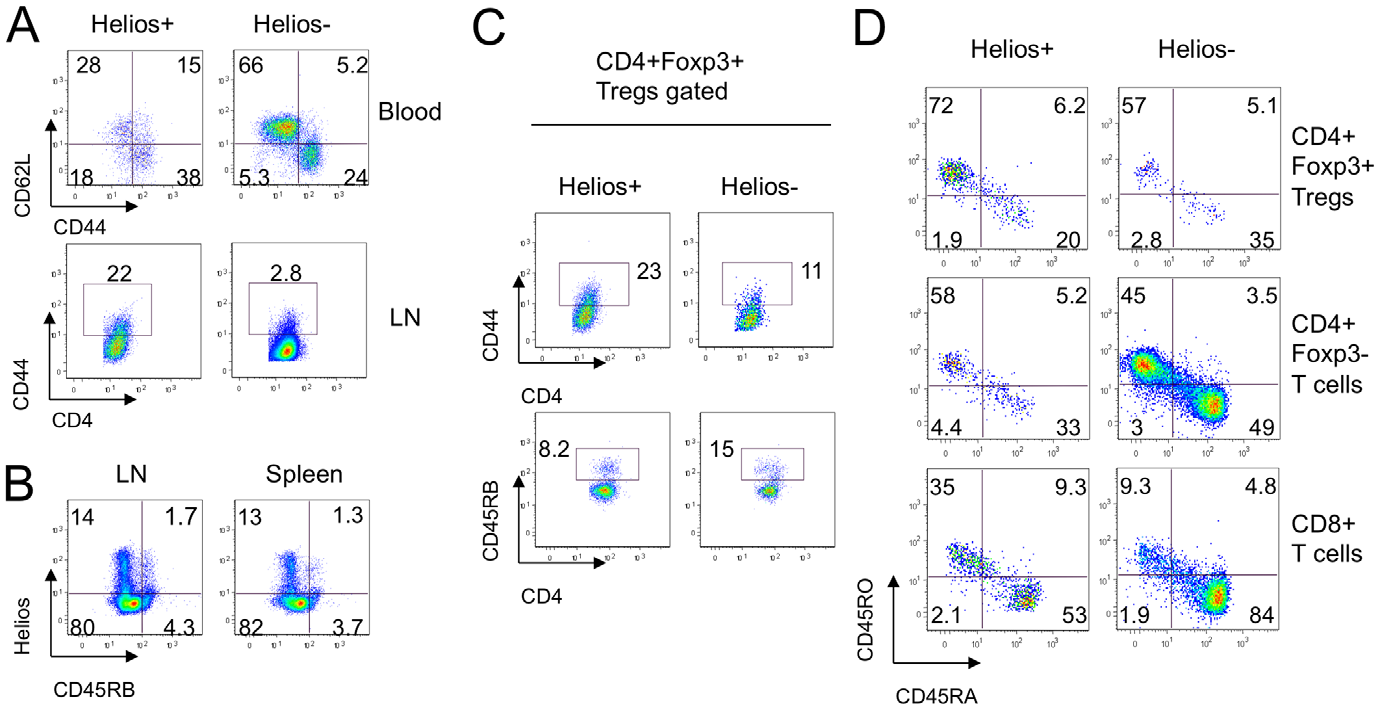
Helios- cells appeared more naïve than Helios+ T cells. (A) Expression of CD62L and CD44 in Helios+ (left column) and Helios- (right column) gated CD4+ cells in murine blood (top) and lymph nodes (second row). (B) Negative correlation between Helios and CD45RB expression in murine lymph nodes and spleen. (C) Expression of CD44 and CD45RB in Helios+ and Helios- gated Tregs in murine lymph nodes. (D) Maturation phenotype in human Tregs (top row), CD4+ Foxp3- Teffs (middle) and CD8+ T cells (bottom row) gated on Helios+ (left column) and Helios- (right column) subsets. Data are representative of 4 experiments.

Flow cytometric analysis showed murine CD4+ Helios- T cells were mostly naïve CD62L+ CD44- cells, whereas CD4+ Helios+ T cells were enriched for memory (CD62L+ CD44+) or effector (CD62L- CD44+) phenotypes (**Figure 2A**). These data suggested that Helios might be induced by T cell activation. Indeed, Helios+ cells largely lacked expression of CD45RB, a CD45 isoform of naïve cells (**Figure 2B**). Since iTreg undergo T cell receptor (TCR) stimulation during conversion, they are less naïve in phenotype than nTregs. Assessment of markers of cell maturation using murine CD4+Foxp3+ Tregs gated into Helios+ or Helios- subsets showed that murine CD4+ Foxp3+ Helios- Tregs had twice as many naïve CD45RB+ cells, and only half as many CD44+ effectors/memory cells, as Helios+ Tregs (**Figure 2C**). Human PBMC showed a similar pattern with Helios+ Tregs (CD4+Foxp3+) or conventional CD4+ Foxp3- T cells and CD8+ T cells being enriched for mature CD45RO+ CD45RA- cells, whereas Helios- populations exhibited the more naïve CD45RO- CD45RA+ phenotype (**Figure 2D**). These data support the concept that Helios may be a marker of T cell activation.

### Helios expression upon T cell activation

**Figure 3:**
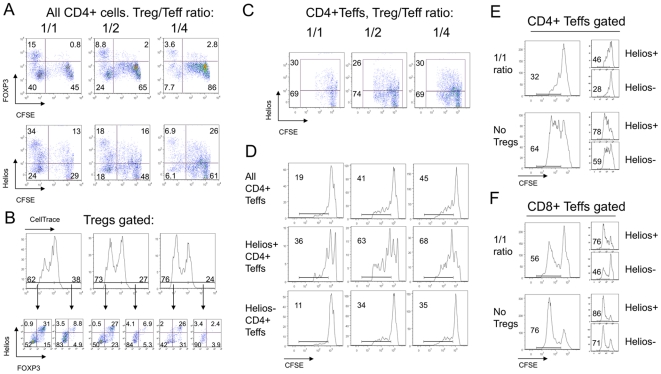
Upregulation of Helios expression by Teff cells in suppression assays. (A) Analysis of CD4+ gated murine Tregs and Teffs after 3 days of suppression assay showed that while Tregs gradually lose Foxp3 (top) and Helios (bottom) expression, a substantial proportion of Teff cells upregulate Helios (bottom), but not Foxp3 (top) expression; CFSE-negative gates show Tregs. (B) Treg division during suppression assays correlated with Helios and Foxp3 co-expression, while non-dividing Tregs lost both markers. Upper row: gating of dividing and non-dividing Tregs, with numbers showing % of Treg divisions at 1/1 (left), ½ (middle) and ¼ (right) ratios; arrows point to Helios and Foxp3 co-expression in corresponding divided and non-divided Tregs subsets through the same 1/1 to ¼ Treg/Teff ratios. (C) Tregs do not induce Helios expression in Teffs since CFSE+ CD4+ Teffs have similar Helios expression despite differing numbers of Tregs (1/1, ½ and ¼ ratios shown). (D) Analysis of Teffs gated into Helios+ and Helios- subsets showed Helios+ Teffs have a higher division rate and greater resistance to Treg suppression than Helios- mouse Teffs. Human Helios+ CD4+ (E) and Helios+ CD8 (F) Teffs showed higher divisions than Helios- cells regardless of Treg presence or absence, and Helios+ responder cells were more resistant to suppression than Helios- responders. Data are representative of 3 experiments.

We tested whether Helios expression is a marker of T cell activation by studying Helios expression in Tregs and T-effector cells (Teffs) during standard *in vitro* Treg suppression assays. In these assays, Teffs are stimulated using CD3 mAb and antigen-presenting cells (APC), in the presence of varying proportions of Tregs, for 3 days (murine cells) or 4 days (human cells). To monitor their divisions during each assay, Teff cells were labeled with CFSE (carboxyfluorescein succinimidyl ester). Numerous murine CFSE+ CD4+ Teffs acquired a Helios+ phenotype, and by day 3, 25–30% of mouse Teffs were Helios+ (**Figure 3A & 3C**); this was an ∼6-fold increase in Helios expression compared to freshly isolated CD4+CD25- cells (**Table 1**). As bead-isolated CD4+ CD25- Teffs could be contaminated by small numbers of Treg cells that could divide and cause an increase in Helios+ T cells, we assessed expression of Foxp3 and Helios by Teffs immediately post-isolation and after suppression assays. Initially, bead-isolated Teffs had about 3% of Foxp3+ and 6% of Helios+ cells (data not shown). After the suppression assay, Foxp3+ expression in CFSE+ murine cells, caused by contaminant Tregs, did not change (**Figure 3A** **, upper row**), whereas Helios expression increased markedly (**Figure 3A** **, lower row**). This finding suggested that murine CD4+ Teffs could acquire expression of Helios, but not Foxp3, under the conditions of TCR stimulation in a Treg suppression assay.

Next, we assessed Foxp3 and Helios expression as a function of Treg proliferation. To study division of Tregs and Teffs in parallel, we labeled CD4+ CD25- Teffs with CFSE, and CD4+ CD25+ Tregs with “*CellTrace*”, a label with similar dye dilution characteristics to those of CFSE, albeit with a different emission frequency on flow cytometry. This enabled us to separate Treg proliferation signals from dividing effector T cells. We found that Tregs had a multiple division peaks, with more at the lowest Treg to Teff cell ratios, ranging from 62% of dividing cells in 1∶1 (**Figure 3B** **, upper row, left**) to 76% in 1∶4 Treg to Teff ratios (**Figure 3B** **, upper row, right**). By comparing dividing and non-dividing Treg, we found that only proliferating Tregs were able to keep Foxp3 and co-express Helios, while non-dividing Tregs lost both molecules almost completely (**Figure 3B** **, lower row**). To assess whether Tregs can induce Helios expression in Teffs as a part of a putative suppressive mechanism, we compared Helios expression in CD4+ CFSE+ Teffs at different Treg to Teff cell ratios. We found that Helios expression by Teff cells did not depend upon the proportions of Tregs added (**Figure 3C**).

**Figure 4:**
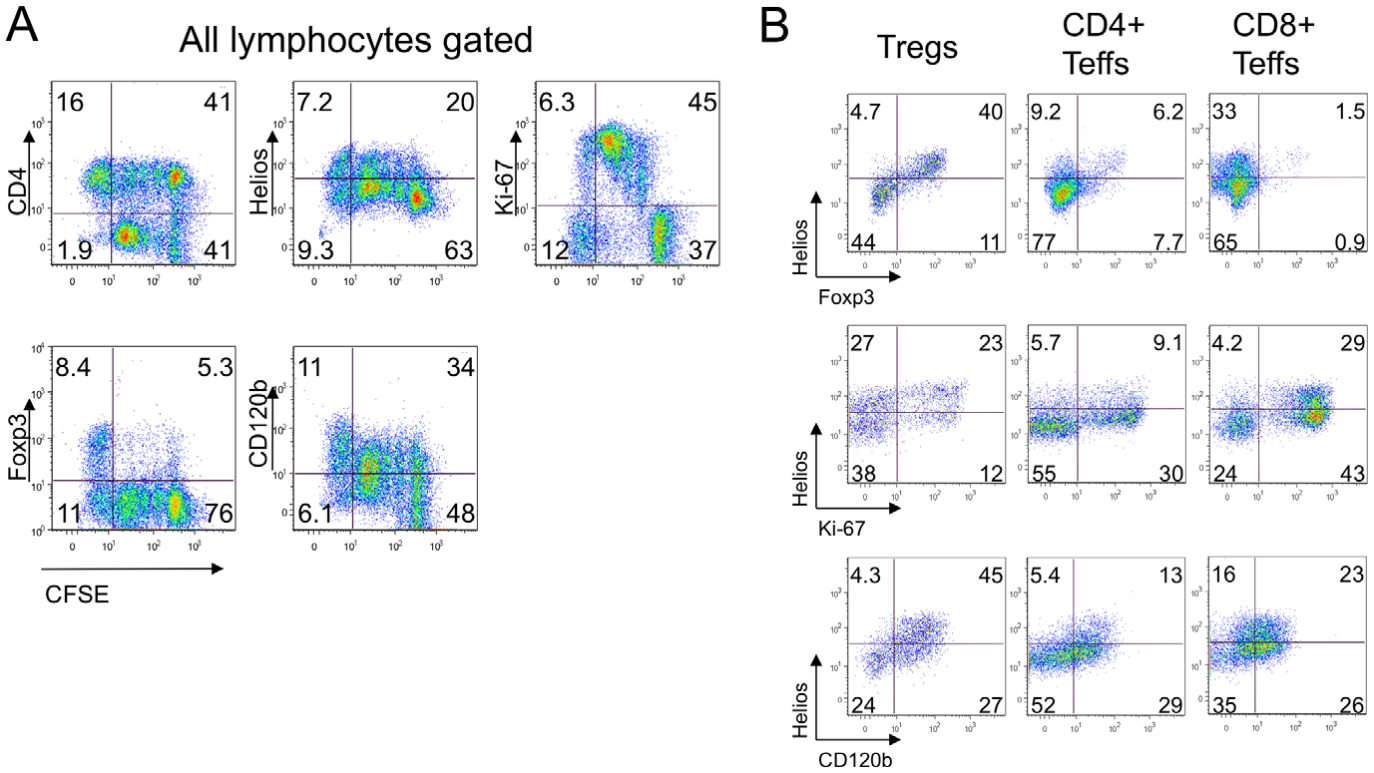
Helios expression in suppression assay is associated with cell activation and division. (A) After suppression assays, human Tregs and responders PBMC cells (1/2 Tregs/responders ratio) were stained for CD4, Helios, Ki-67, Foxp3 and CD120b; gating of live lymphocytes and co- expression of listed markers with CFSE divisions peaks shown, cells in CFSE-negative gate are Tregs. (B) Helios+ Tregs but not CD4+ and CD8+ responders showed co-expression of Helios and Foxp3 (top row). Helios+ cells showed more dividing (middle) and activated (bottom) phenotypes throughout all T cells subsets, including Tregs (left), CD4+ (middle) and CD8+ (right) cells. Data are representative of 2 experiments.

Although we showed that highly activated Teffs can become Helios+ under the conditions of a Treg suppression assay, it remained unclear whether this Helios+ phenotype reflected a type of negative-feedback loop preventing hyperactivation, or was primarily related to cell activation, akin to CD25 expression. Therefore, we defined Helios+ and Helios- Teff subsets and analyzed separately their cellular division rates. As seen in Figure 3D, Helios+ Teffs exhibited much higher division throughout all Treg to Teff ratios. Therefore, Helios+ Teffs are a subset of highly activated and dividing effector T cells that are relatively resistant to Treg suppression. Likewise, human CD4+ CD25-, and even CD8+ T cells acquired Helios during a Treg suppression assay (**Figure 3E & 3F**, and **Figure 4A & B**). As with murine cells, human CD4+ and CD8+ T cells that acquired Helios were more resistant to suppression and had higher rates of cellular division (**Figure 3E & 3F**).

To investigate the relationship of Helios expression with cell division and resistance of Teff cells to Treg suppression, we studied co-expression of Helios with Ki-67, a marker for cellular proliferation, as well as CD120b (TNFR2, the type 2 TNF receptor), a proposed marker for Teff cells resistant to suppression, under the conditions of a human Treg suppression assay (**Figure 4A**). Most dividing Ki-67+ Tregs were Helios+ (**Figure 4B** **, left column**). The majority of Helios positive CD4+ and CD8+ T cells were negative for Foxp3, despite some acquired Foxp3 expression in CD4+ (14%) and CD8+ (2.4%) cells (**Figure 4B** **, upper row**). Most Helios+ Teffs were highly proliferative (**Figure 4B** **, middle row**). Finally, CD120b expression correlated with Helios expression in Tregs, CD4+ and in CD8+ Teffs (**Figure 4B** **, lower row**), confirming the activated phenotype of Helios+ Tregs and resistance to suppression of Helios+ T effectors. These data further supported the hypothesis that Helios may be a marker of activated and dividing T cells.

**Figure 5:**
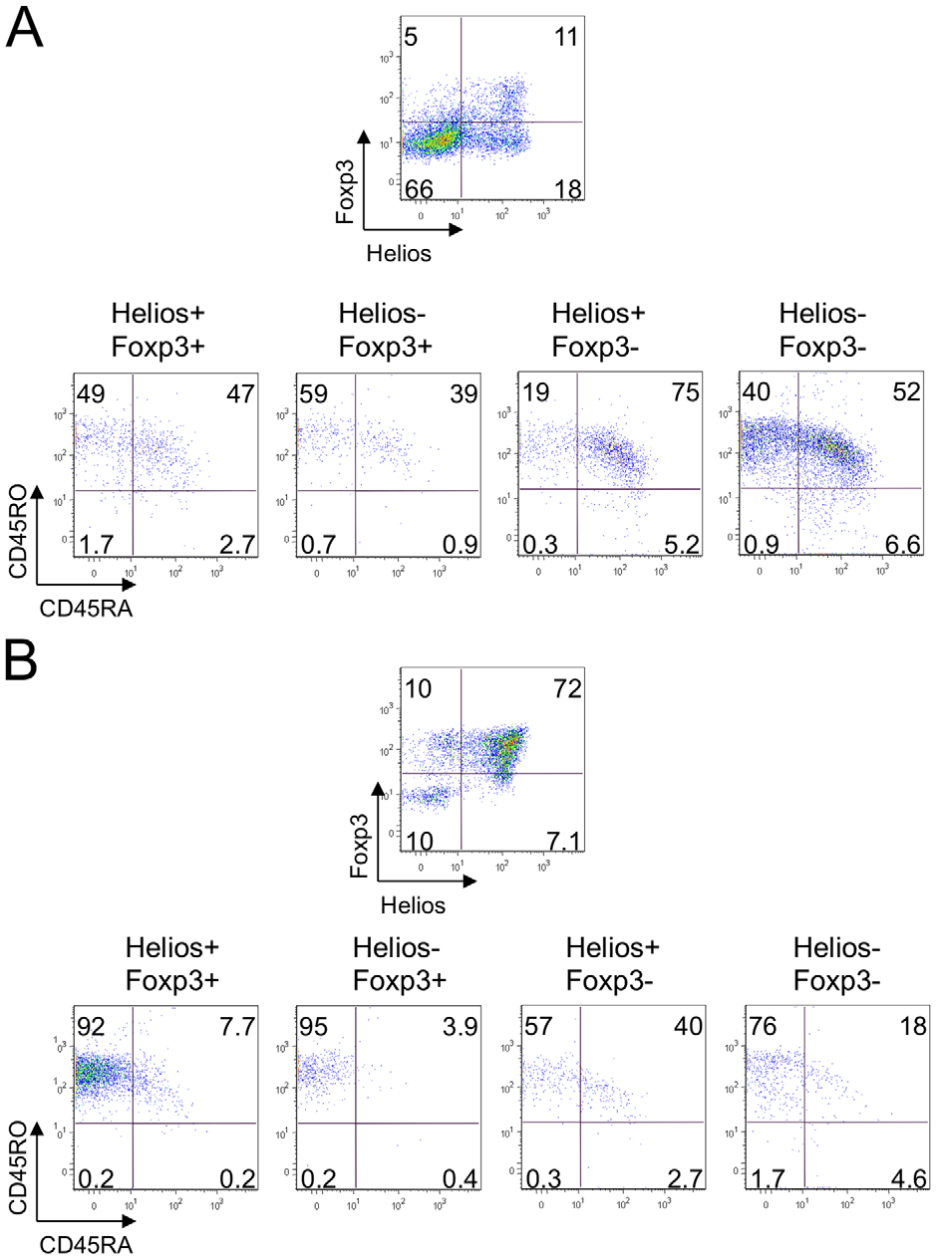
Helios is associated with an activated non-mature phenotype in suppression assays. After suppression assays (1/2 Treg/responder ratio), human CD4+ Teffs (A) and Tregs (B) were divided into 4 subsets according to Helios and Foxp3 co-expression. Helios+Foxp3- cells had the least mature and the most activated phenotypes in human CD4+ Teffs (A) and Tregs (B) in suppression assays, while Foxp3 expressing cells were enriched for mature CD45RO+ CD45RA- subsets. Data are representative of 2 experiments.

To further substantiate this concept, we investigated the relationship between Helios and Foxp3 expression and maturation/activation phenotype in T cells under the condition of a Treg suppression assay. We divided human CD4+ Teffs (**Figure 5A**), CD8+ (not shown) and Tregs (**Figure 5B**) after suppression assay into four subsets: Helios+ Foxp3+, Helios- Foxp3+, Helios+ Foxp3- and Helios- Foxp3- cells, and analyzed CD45RA, CD45RO and CD62L expression. We observed that human T cells gradually lost CD45RA expression during cell divisions, developed CD45RO expression and mostly kept CD62L expression, forming three subsets: fully maturated CD62L+ (or low) CD45RO+ CD45RA- cells, naïve CD62L+ CD45RO-CD45RA+ cells and activated CD62L+ CD45RA+ CD45RO+ cells which have already acquired CD45RO marker, but still kept CD45RA and CD62L expression. Hence, two Foxp3+ subsets, independent of Helios expression, were enriched for mature effector and memory cells, while Helios+ Foxp3- cells were composed of highly activated CD45RA+ CD45RO+ cells, and double negative Foxp3-Helios- subsets were enriched for naïve cells (**Figure 5**). The same patterns were observed for CD8+ effector T cells (not shown). These data further supported our hypothesis of Helios upregulation upon T cell activation.

### IL-2 enhances Helios expression in stimulated T cells without acquisition of a Treg phenotype

**Figure 6:**
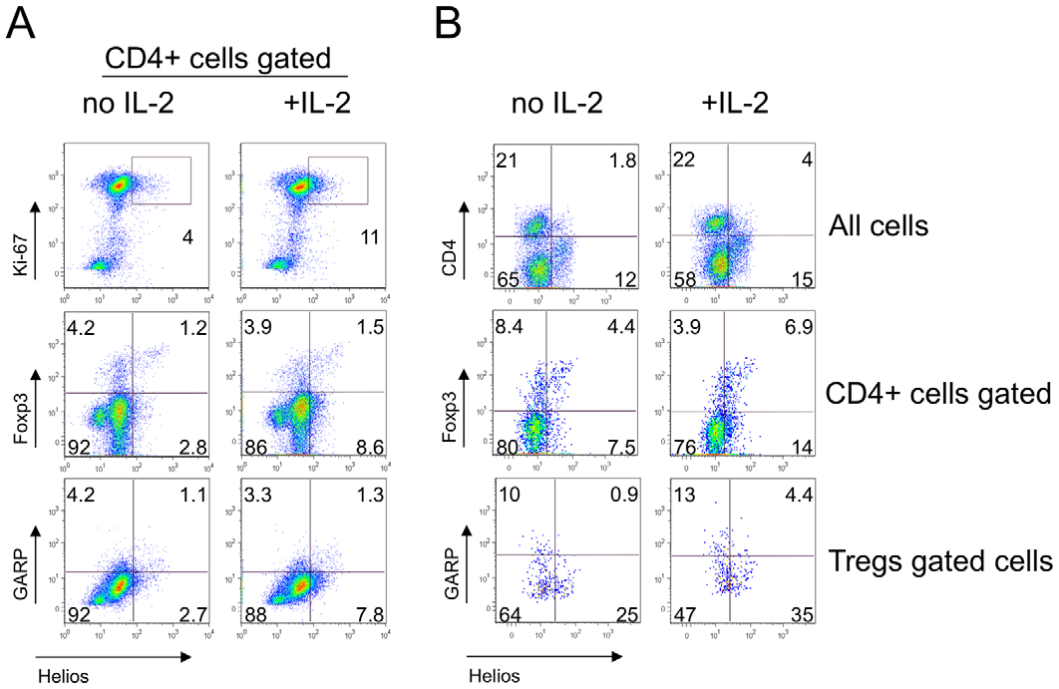
IL-2 increased Helios expression in Ki-67+ Foxp3- GARP- cells. Murine MNC from lymph nodes and spleen (A) and human PBMC (B) were stimulated with CD3/CD28±IL-2 for 24 hrs and stained for CD4, Foxp3, Helios, Ki-67 and GARP. IL-2 addition led to a slight increase in expression of Helios by Ki-67+ (A, top) CD4+ and CD4- (B, top) and Foxp3- cells (A, B, middle rows). Helios expression was not associated with GARP expression in CD4+ gated (A, bottom) or Foxp3+ gated (B, bottom) cells.

Recently, a new Treg-associated surface marker called GARP was suggested to discriminate “true” suppressive Tregs from activated CD25+ CD127^low^ Foxp3+ CTLA-4+ expressing Teff cells [23]. To induce GARP expression, cells need to be activated with anti-CD3ε and anti-CD28 in the presence of IL-2 for at least 24 hours. We studied Helios and GARP co-expression in mice and human lymphocytes, and assessed whether Helios expression could be enhanced by the addition of IL-2. We found that IL-2 led to a moderate increase in Helios expression from 4 to 11%, and from 12 to 21%, in murine and human CD4+ T cells, respectively (**Figure 6A** **, upper row and 6B, middle row**). Of note, Helios+ cells were also Ki-67+. At the same time, IL-2 did not increase Foxp3 expression, and the IL-2 treated Helios+ subset was enriched with Foxp3- cells (**Figure 6** **A & 6B, middle rows**). The addition of IL-2 led to a minor increase in GARP expression, perhaps due to short time and sufficient level of internal IL-2 from non-Treg cells. However, GARP did not correlate with Helios expression in CD4+ or in CD4+Foxp3+ cells (**Figure 6A, B** **, lower rows**), with or without addition of IL-2. Restricting the incubation period to 24 hours allowed detection of increases in Helios expression that were independent of cell division, and again underlined the association of Helios expression with cellular activation.

### Helios expression is not associated with Treg lineage commitment

**Figure 7:**
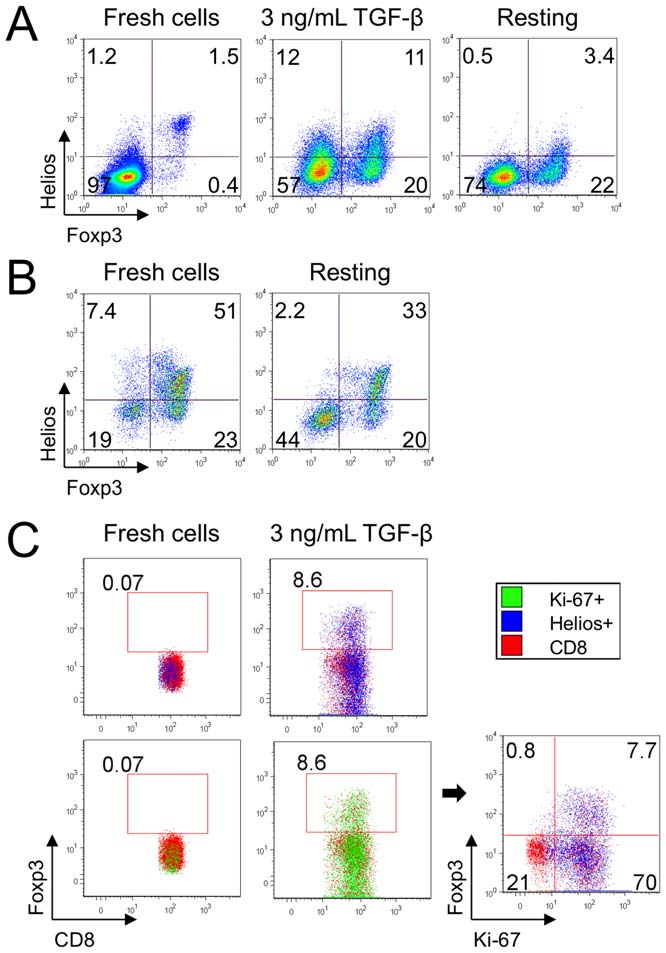
Helios is not associated with Treg lineage commitment in mice. (A) Murine CD4+ CD25- Teffs stimulated with CD3/CD28 mAbs, IL-2 and TGF-beta to become iTregs upregulated Helios in both Foxp3+ and Foxp3- subsets (middle). Withdrawal of TCR stimulation and TGF-beta led to loss of Helios expression (right). (B) Murine Tregs after isolation (left) and after 4 d with IL-2 but without stimulation (right). In resting conditions Helios expression decreased, and Foxp3 expression by Tregs decreased mainly in the Helios+ subset. (C) Induction of Foxp3 in mouse CD8+ cells in the same conditions as in (A). Foxp3+ converted CD8 cells co-expressed Helios and Ki-67.

Given that Helios and Foxp3 were both elevated in the fraction of dividing Tregs in the suppression assay (**Figure 3B** **&** **4B**), we considered whether Helios might be important for stabilizing the Foxp3+ Treg phenotype. We stimulated Teff cells to become Foxp3+ iTreg by CD3ε/CD28 co-stimulation, in the presence of 3 ng/mL TGF-beta, for four days; these sub-optimal conditions ensured that some Foxp3- cells remained available for comparisons. Approximately 30% of CD4+ CD25- Foxp3- T-cells became Foxp3+ and 22% upregulated Helios, in both Foxp3+ and Foxp3- subsets, with a higher percent of Helios+ cells in iTregs. Next, we removed CD3ε/CD28, as well as TGF-beta stimulation, and cultured the cells in IL-2 (25 U/ml) for an additional 4 days. We found that the removal of stimulation resulted in a decline of Helios expression (**Figure 7A**). The same finding was seen when freshly isolated CD4+CD25+ nTreg were incubated in IL-2 (25 U/ml, 4 days) without stimulation. The fraction of Helios+ cells declined sharply, and the Foxp3+ decrease occurred mostly among the Helios+ cell subset since the proportion of Helios-Foxp3+nTregs changed slightly, from 23 to 20% (**Figure 7B**). Of note, the induction of Helios was seen in CD8+ as well as CD4+ T cells. We induced Foxp3+ in CD8+ T-cells by stimulating them under the same conditions as in **Figure 7A**, and found that both Helios and Ki-67 were upregulated together in stimulated CD8+ cells (**Figure 7C**), similar to what we observed with human CD8+ cells in the Treg suppression assay.

### Helios and Ki-67 are co-expressed by murine T-cells *in vivo*

**Figure 8:**
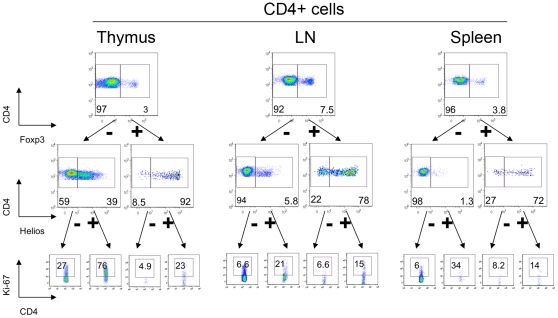
Helios+ expression correlates with Ki-67 expression *in vivo*. Murine thymic (left), lymph node (middle) and splenic (right) CD4+ gated cells were divided into Foxp3+ Tregs and Foxp3- cells (top row), and then divided into Helios+ and Helios- subsets (middle), and Ki-67 expression within each subset was analyzed (bottom).

Given our observations that Helios was associated with cellular activation and division *in vitro*, we tested cells from normal murine thymi, spleens and lymph nodes for co-expression of Helios and a marker of cellular proliferation, Ki-67 (**Figure 8**). We found that compared to cells from the spleen and lymph nodes, the thymus was highly positive for CD4+ T cells expressing both Helios and Ki-67. Next, we investigated the Ki-67-positive fractions of CD4+CD8-Foxp3/Helios subsets. We found that Helios+ cells were enriched for Ki-67 expression throughout each subset of CD4+ (**Figure 8A**) and CD8+ T cells (not shown).

### Foxp3+ Helios- cells in the thymus do not originate from peripheral iTreg

**Figure 9:**
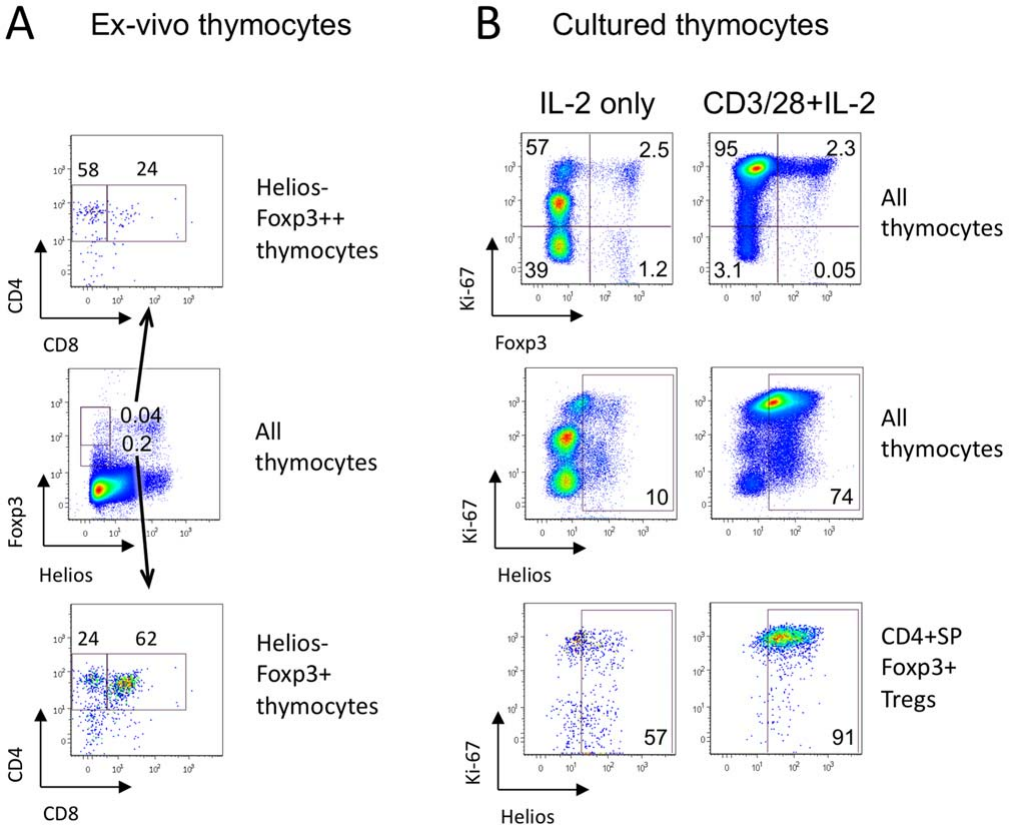
Helios expression in thymocytes depends on cell stimulation and not origin. (A) Helios- Foxp3+ subsets were gated within murine thymic cells, and CD4 and CD8 distribution was analyzed. More than half (bottom) of Foxp3+ Helios- cells and 24% (top) of Foxp3^hi^ Helios- cells in the thymus were immature CD4+ CD8+ DP thymocytes. (B) Murine thymic cells were incubated for 3 days with IL-2 only (non-stimulated, left column) or were stimulated with CD3/CD28 mAbs and IL-2 (stimulated, right column). Ki-67 expression (top) and Helios expression (middle) in non-stimulated cells decreased markedly; and Helios expression in thymic Tregs (CD4+ SP Foxp3+ cells) decreased from 91% to 57% (bottom).

To study the possible origin of Foxp3+ Helios- cells in the thymus, we tested expression of CD4 and CD8 within this population. Of note, 62% of Foxp3+ Helios- cells and 24% of Foxp3^high^ Helios- cells exhibited the double-positive CD4+CD8+ stage of thymic development, which argued against the idea that Foxp3+ Helios- cells are recirculating iTregs cells that were induced in the periphery (**Figure 9A**). To study Helios expression in thymocytes in resting conditions, we divided freshly isolated cells into 2 portions, and cultured them with IL-2±CD3/CD28 stimulation for 3 days. Despite the presence the thymic APC cells in these cultures, unstimulated cells lost almost half their Ki-67 expression (59.5% vs. 97% in stimulated cells, and vs. 90% in fresh thymocytes) and most Helios expression (10% vs. 74% in stimulated cells, and vs. 27% of Helios+ cells in fresh thymocytes) (**Figure 9B** **, top and middle rows**). However, in unstimulated conditions, 68% of Foxp3+ cells were still Ki-67+ (**Figure 9B** **, top, left**), suggesting that Tregs were not as dependent upon TCR stimulation to divide as conventional T cells. In spite of this, CD4+ SP Foxp3+ Tregs markedly lost Helios expression: 57% Helios+ cells in non-stimulated thymic Tregs vs. 91% in stimulated Tregs (**Figure 9B** **, bottom**) and vs. 90% in ex-vivo thymic Tregs (data not shown).

In these studies, we can exclude the possibility of overgrowth of thymic Helios+ nTregs (which stopped division) by peripheral Helios- iTregs (which continued dividing) for multiple reasons. First, the proportions of Helios+ cells in gated dividing Ki-67+ and non-dividing Ki-67- CD4+SP Foxp3+ Tregs in resting conditions was the same, 57%, and, respectively, the proportions of Ki-67+ dividing cells in Helios+ and Helios- subsets of CD4+ SP Foxp3+ Tregs in resting conditions were the same, 62% and 63%. Second, in resting conditions 21% of all Ki-67+ dividing Foxp3+ Helios- thymocytes were immature DP CD4+CD8+ cells, the type of cells, which don't originate in peripheral tissues. Third, the proportion of cells with compromised membranes (Dead+ cells within the lymphocyte gate as determined using the LIVE/DEAD Fixable Dead Cell Stain Kit) in non-stimulated conditions was the same as in stimulated cells; these consisted of a negligible number of Foxp3+ cells (0.2%), and half of them (47%) were Helios-. Lastly, the proportion of Foxp3+ cells was similar in resting and in activated conditions of thymic cells (**Figure 9** **B, top**). Hence, the viability of Helios+ and Helios- cells in resting conditions was the same, and loss of Helios+ Tregs could not be explained by preferential survival of a Helios- subset. As a result, thymic Tregs cells are Helios+ due to their activation/division state and not their site of origin, and this Helios+ phenotype is changeable even for thymocytes.

### Helios expression of Helios by *in vitro* expanded Tregs and in clinical transplant recipients

Our lab undertakes immune monitoring of adult and pediatric liver transplant recipients receiving standard triple therapy of calcineurin inhibitor, corticosteroids and azathioprine. In adults, we collect blood pre-transplant, plus in the first week post-transplant and at 3 months, whereas in children, blood is collected only from patients with stable graft function and no episodes of rejection during the prior 6 months. In both projects, Tregs are isolated using magnetic beads and tested in Treg suppression assays. Within these samples, no correlation between Treg expression of Helios and donor age was observed (**Table 2**). We likewise studied Helios expression in control healthy donors, aged 25–40 years. Interestingly, the highest Helios expression was seen with expanded Tregs from a healthy male donor (**Table 2**). While observations involving expanded Tregs might be explained in different ways, including variation in Foxp3 expression or the period of stimulation/activation of these cells, other samples had comparable Foxp3 expression (60%–70%) and should have correlated with age if Helios expression reflected a thymic origin of Tregs.

**Table 2:** Helios expression in Tregs in different ages.

Sample	Age	Gender	% Helios expression in isolated Tregs
01 liver Tx	8	F	71
02 liver Tx	11	F	33
03 liver Tx	13	F	42
04 liver Tx	15	F	29
01 donor	24	F	14
05 liver Tx	32	M	50
02 donor	33	F	56
03 donor	25-40	M	65
04 donor	25-40	M	79
06 liver Tx	57	M	44
07 liver Tx	59	F	61
05 donor – expanded Tregs	25-40	M	90

**Figure 10:**
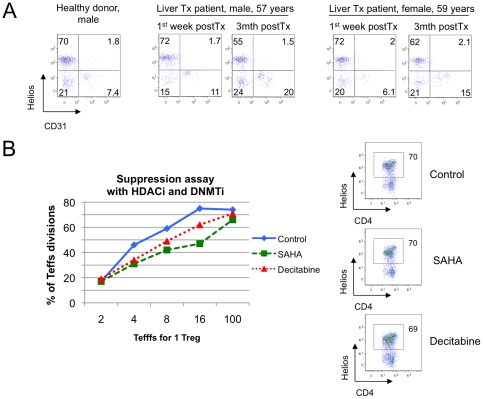
Helios expression by human Tregs did not correlate with CD31 or suppressive function. (A) Two liver transplant patients underwent serial analysis of Treg suppressive function, Foxp3 methylation and cell phenotype. At 3 mths post-transplant, Tregs had increased thymic output of CD31+ Tregs and reduction of Helios+ Tregs; Helios was not co-expressed with CD31. (B) Tregs, CD4+CD25- Teffs and CD4-depleted APC from healthy donor were used in suppression assays, with or without treatment with a DNMTi (Decitabine) or HDACi (SAHA). Enhancement of Treg suppression by either agent (left) was not accompanied by change in Helios expression (right).

Foxp3 demethylation within the TSDR (Treg-specific demethylated region) of human Tregs distinguishes nTreg from iTregs produced *in vitro* or from activated, transiently Foxp3+ Teff cells [24], and might provide additional light on the relationship of Helios expression and FOXP3. We therefore examined Helios expression, Foxp3 demethylation and Treg function using cells isolated from serial blood samples of two adult liver transplant recipients. Compared to levels in the first week post-transplant, Helios expression by patient Tregs was decreased at 3 months post-transplant (**Table 3**). While this may be consistent with increased iTregs populations, other data in Table 3 argue against this viewpoint. First, liver transplantation caused an increase, rather than a decrease, in Treg expression of CD31 (**Figure 10A**), a marker of recent thymic emigrants, followed by increase of naïve CD45RA+ subset (1^st^ patient) or a decrease of CD45RO+ mature subset of Tregs (2^nd^ patient). Second, we noted decreased Foxp3 demethylation in one patient, while the other exhibited increased Foxp3 demethylation. Third, Treg suppressive function, shown in Table 3 as area-under curve (AUC) data [25], improved in the female patient with autoimmune liver pathology, along with increased Foxp3 demethylation, and declined in the male patient with alcoholic liver disease, along with decreased Foxp3 demethylation. Such data were consistent with the correlation between Foxp3 demethylation and Treg function, whereas no comparable link was found between Helios expression and thymic Treg production, Foxp3 methylation or suppressive function. Lastly, we tested assess whether Helios expression in Tregs was associated with changes in normal healthy donor Treg suppressive function induced by pharmacologic treatment with a histone/protein deacetylase inhibitor (HDACi) or a DNA methyltransferase inhibitor (DNMTi) [25]. We found that treatment with an HDACi (SAHA) or a DNMTi (Decitabine) increased human Treg suppressive function *in vitro*, but Helios expression in Tregs remained unchanged (**Figure 10B**).

**Table 3:** Helios expression, FOXP3 demethylation, maturation phenotype and Treg suppressive function.

Patient, linical data		% Helios expression	% Unmethylated FOXP3 in TSDR	Suppressive function, AUC		% CD31 expression	% CD45RA+ D45RO-expression	% CD45RO+ D45RA-expression
				CD4+ esponders	CD8+ esponders			
Male, 57 yrs, alcoholic liver disease	1 wk post Tx	74	66	139	97	12	17	22
	3 mths post Tx	57	46	105	0	22	27	21
Female, 59 yrs,Primary biliary cirrhosis	1 wk post Tx	74	42	14	5	8	7	88
	3 mths post Tx	64	50	35	39	17	7	82
Healthy donor, male, 25-40 yrs		72	76	204	No data	9	27	65

## Discussion

Helios is a highly conserved transcription factor that has 97% sequence homology between humans and mice [26]. Previous studies showed that constitutive expression of Helios in T cells led to inhibition of thymic T cell development [27], while a mutation negating DNA binding ability led to T cell leukemia in both mice [27] and humans [28]. However, full knockout of Helios in mice did not significantly affect T cell development, including that of Foxp3+ Tregs [29], suggesting the compensatory potential of residual Ikaros family members. Recently, Thornton et al [21] reported that Helios expression in human and murine Tregs could discriminate thymic-derived nTregs from iTregs, as nTregs co-expressed Helios but iTregs developing *in vitro* or *in vivo* did not. In a subsequent letter, Verhagen and Wraith, in studies of iTreg induction from Teff cells of Rag-deficient Tg4 transgenic mice Teffs to induce Tregs, suggested that the method of *in vitro* activation (i.e. the presence or absence of APC), rather than the thymic vs. peripheral origin of Foxp3-expressing cells, determined Helios expression [22]. These conflicting data may reflect limitations of each model. Thus, *in vitro* induced murine Tregs do not have a stable iTreg phenotype and lose Foxp3 after TGF-beta withdrawal, while human iTregs generated this way may be unstable and/or poorly suppressive [24], [30], [31]. Also, given the limited knowledge of the biological role of Helios in T cells, use of immunodeficient mice could introduce additional confounding variables. We therefore focused our studies primarily on Helios expression in T cells present under natural conditions in untreated, non-genetically altered, healthy young C57BL/6 mice, as well as in 25–40 year old healthy human donors.

Over the last decade, numerous Treg subtypes were identified [32]. Some of them, such as CD45RA+CD45RO-CD62L+ human Tregs, have a naïve phenotype comparable similar to naïve conventional T cells, while others are more restricted to Tregs. Such Treg selectivity may be based upon quantitative differences such as high expression of CD25 and low expression of CD127, or by the functional significance of the protein to Treg biology. Examples of functionally significant Treg proteins include CTLA-4 [9], CD39 [33], GARP [23], [34], and CD120b (TNFR2) [35], though these molecules are also expressed by other cells, reducing their utility and typically requiring multiparameter analysis. Thus, TNFR2 was shown to be up-regulated by Tregs with the greatest suppression activity in both humans [35] and mice [36], but also to be associated with increased resistance to suppression when expressed by activated Teff cells [37]. In the current work, Helios expression was not restricted to CD4+ cells, and within the CD4+ fraction, Helios expression was not restricted to Foxp3+ Tregs. However, we did find a common association between high Helios expression and a set of Treg-known markers including Foxp3, CD25, CD39, CTLA-4 and low levels of CD127. As CD31 expression has become recognized as a marker of recently emigrated, thymic-derived Tregs [15], [38], [39], we expected that Helios+ CD4+ T cells (including Tregs) would co-express CD31. However, we were surprised to find that CD31 expression was inversely associated with Helios expression in Tregs. CD31 is cleaved from the surfaces of human T cells upon TCR activation [14], so we also investigated the maturation phenotype of Helios+ and Helios- cells using standard markers of CD45 isoforms and CD62L expression. We found that Helios- T cells (Tregs, CD4+Foxp3- cells, CD8 cells) in mice and humans were enriched for naïve phenotypes, while Helios+ cells showed more mature phenotypic characteristics. Since iTreg require TCR-induced activation for their development, these data were not consistent with the concept that naive nTregs are primarily Helios+ and that iTregs are primarily Helios- cells.

Treg suppression assays provide the key activating and co-stimulatory signals to analyze Treg functions under standardized conditions. Using this *in vitro* system and analyzing the characteristics of working Tregs and associated dividing responder cells, we showed that Helios could be induced within responder cells (CD4 or CD8 Teffs) even without addition of TGF-beta or IL-2. Murine CD4+ cells that upregulated Helios+ did not express Foxp3, making unlikely the possibility that observed Helios expression came from contaminant Tregs. In human Treg suppression assays, some Teffs upregulated Foxp3 to an intermediate level, but Helios expression exceeded Foxp3 expression significantly. Moreover, human CD8 effector cells also upregulated Helios expression under the same conditions. Helios was induced most prominently among highly dividing cells, which are most activated and resistant to Treg suppression. These data further substantiated our hypothesis that Helios expression simply reflected T cell activation and proliferation.

While examining Helios expression within Tregs present in suppression assays, we noticed that Helios most prominently expressed by dividing Tregs, analogous to the pattern seen in Teff cells. Conversely, non-dividing Tregs rapidly lost Foxp3 and Helios expression. This observation is consistent with the impaired suppressive activity of human Treg seen after Helios knockdown [40], though the phenotype and proliferation rate of Helios knockdown Tregs, compared to unmanipulated or control siRNA Tregs, were not described. On the other hand, Thornton et al [21] noted that ex vivo expanded human Tregs treated with Helios siRNA had normal suppressive function. However, their experimental setup was based on use of cells expanded for 14 days with high levels of stimulation plus IL-2 supplementation. While the exact role of Helios in T cells remains elusive, yet another member of the Ikaros family, Eos, was shown to mediate Foxp3-dependent gene silencing and was critical for Treg suppressive function [41].

To assess if Helios upregulation *in vitro* was further enhanced by IL-2, and to analyze GARP expression, we stimulated murine lymph node and spleen cells, and human PBMC, with CD3ε/CD28 mAbs±IL-2. Helios upregulation was increased by CD3/CD28 stimulation as soon as 24 hours, with a moderate increase upon addition of IL-2, while Foxp3 expression remained unchanged. Helios expression did not correlate with GARP expression in CD4+ T cells or Tregs, suggesting Helios expression might be an activation marker and, indeed, Helios+ cells co-expressed the Ki-67 proliferation marker. In addition, since the short time incubation precluded division of thymic Helios+ Tregs cells, these data indicated that Helios upregulation occurred in previously Helios- cells. We also tested the effects of TGF-beta to promote iTreg development and found Helios was upregulated in both Foxp3+ and Foxp3- subsets. Withdrawal of stimulation led to a rapid decline of Helios in Foxp3+ Helios+ and Foxp3- Helios+ subsets, while Foxp3 expression was maintained in the Helios- iTregs subset. The fast down-regulation of Helios upon removing TCR stimulation, or the absence of stimulatory signals, might explain the effect observed by Thornton et al. whereby *in vitro* induced iTregs remained Helios- [21]. Furthermore, the persistence of remaining APC co-stimulation might explain Helios+ Foxp3+ co-expression, as observed by Verhagen and Wraith [22].

We next sought *in vivo* evidence of Helios expression. Analysis of murine lymphoid tissues showed a close association of Helios and Ki-67 expression. Our data help explain the marked Helios expression of Tregs, since thymic CD4+ and CD8+ T cells have higher Ki-67 expression than their peripheral counterparts. Thornton et al. [21] also showed an absence of Helios- Foxp3+ in newborn mice until 7 days of life, and Foxp3+ Helios+ cells expressed a high level of CD44 while Foxp3+ Helios- cells appeared later and remained mostly CD44^low^ naïve for the first weeks. These observations are in line with our hypothesis. Neonatal murine T cells undergo rapid division just after leaving the thymus, in a process termed homeostatic proliferation [42]. Short-term labeling revealed at least 10-fold more murine neonatal cells of both the CD4+ and CD8+ populations within the cycle, as compared with their adult counterparts [43]. Neonatal dividing T cells (as well as Tregs) acquired a CD44+ phenotype, and, we would predict, would become Helios+ as a result of their high proliferation rate. Later, as physiological lymphopenia disappears and homeostatic proliferation declines, the proportion of non-dividing T cells increases, and resting, non-dividing Foxp3+Helios- with naïve CD44^low^ would appear in the periphery. However, thymic cells have a high division rate throughout life, explaining their relatively higher Helios expression in comparison to peripheral cells. Consistent with this concept, we found that in resting conditions thymocytes (including Tregs) lost Helios expression, but that the same cells upregulated Helios, exceeding *in vivo* physiologic level, under strong CD3/CD28 stimulation.

Thornton et al [21] and Fujimoto et al [44] suggested that the small subset of Foxp3+ Helios- cells observed in the thymus comprise iTregs that re-entered the thymus from the periphery. However, we showed that more than half of these cells are immature CD4+ CD8+ double-positive thymocytes. Fujimoto at al. [44] also showed that excessive IL-6 production in IL-6 transgenic mice led to aberrant peripheral T cell activation and increased Helios expression within Foxp3+ Tregs, but normal *in vitro* and *in vivo* induction of iTregs from naïve IL-6 transgenic CD4+ T cells, and no differences in iTreg induction after anti-IL-6R mAb treatment. If Helios- Foxp3+ Tregs were iTregs whose formation were inhibited by increased IL-6, then normal *in vitro* and *in vivo* induction of iTregs from the same naïve IL-6 transgenic CD4+ T cells contradict these *in vivo* observations. This contradiction is resolved if Helios expression is considered as a consequence of the cellular activation shown in these mice. Therefore, the activated phenotype of T cells observed in IL-6 transgenic mice along with increased Helios expression supports the cellular activation, but not the thymic-derived, hypothesis.

We could not find a relationship between age and Helios expression in Tregs samples isolated from children, adults aged 25-40 yrs or elderly persons, which supports the theory that Helios expression may not be related to thymic involution in humans and therefore may not be connected with the thymic origin of Tregs. By serial analysis of Tregs obtained from liver transplant patients, we showed that Helios expression was neither related to Foxp3 methylation nor to Treg suppressive function, though the latter two factors are correlated with each other. Our data also do not contradict recent findings by McClymont et al [45] showing increased frequency of Helios- Foxp3+ IFN-gamma+ Tregs in diabetic patients and increased Foxp3 methylation in Helios- IFN-gamma+ Tregs from healthy donors, since they did not use nTregs in any experiments, but rather, analyzed cells obtained from one particular CD4+ CD127lo/- CD25+ CD45RA+ subset of natural Tregs and then expanded for 2 wks under conditions of high activation and IL-2 supplementation. Finally, we showed that epigenetic changes in Tregs induced by exposure to HDACi and DNMTi correlated with enhanced suppressive abilities, but were not associated with any changes in Helios expression.

In conclusion, Helios can be induced within CD4+ T cells, CD8+ T cells and in Tregs in response to cellular activation, and regresses in the same cells under resting conditions. Based on our data, Helios expression is not a suitable tool to distinguish natural from induced Treg cells.

## Materials and Methods

### Ethics statement

Clinical studies were approved by the Institutional Review Board of the Hospital of the University of Pennsylvania (#810878), and animal studies were approved by the Institutional Animal Care and Use Committee of The Children's Hospital of Philadelphia (approval number #2010-6-561).

### DNMTi (DNA Methyl Transferase inhibitor) and HDACi (Histone deacetylase inhibitor)

We purchased decitabine (5-Aza-2′-deoxycytidine, DNMTi) from Sigma and SAHA (Suberoylanilide hydroxamic acid, pan-HDACi) from Cayman Chemical.

### Human mononuclear cell isolation, Treg expansion and Treg suppression assays

Mononuclear cell-enriched apheresis product was obtained by leukapheresis of healthy volunteer donors (n = 4; 2 females) by the University of Pennsylvania Human Immunology Core. Specimens were collected according to the protocol approved by the Hospital of the University of Pennsylvania Institutional Review Board, and informed consent was obtained from each donor. Human Treg expansion was performed as described [46]; briefly, male donor FACS-sorted CD25hi Tregs were incubated with OKT-3 CD3ε mAb-loaded artificial APC (K64.86) for 12–14 days in the presence of IL-2 and human AB serum. Human Treg suppression assays were performed, and Treg suppressive function calculated as area under the standardized suppression curve (AUC), as described [25]. Briefly, CD4+ CD25+ Tregs isolated by magnetic beads (Miltenyi Biotec) were stimulated with CD3ε mAb-coated microbeads and incubated with CFSE-labeled PBMC at 1∶1 to 1∶16 Treg: PBMC ratios for 4 days. Experiments with Decitabine (125 nM) and SAHA (200 nM) were performed with Tregs, CD4-depleted APC and CD4+ CD25- Teffs, as described [25].

### Human Foxp3 methylation assay

We isolated genomic DNA from CD4+ CD25+ beads-isolated Tregs using Puregene Kit A (Qiagen), according to the manufacturer's instructions. To detect differentially methylated DNA fractions, we exposed the DNA to two restriction enzymes, one methylation-sensitive and the other methylation dependent. We then performed enzymatic digestions according to the manufacturer's instructions (Methyl-Profiler DNA Methylation Enzyme Kit, SABiosciences). Next, we conducted DNA methylation PCR arrays using custom Foxp3 primers (SABiosciences) corresponding to the Treg-specific demethylated region (TSDR) of human Foxp3. Using internal control digests, we calculated the percent of methylated CpG islands in TSDR region of Foxp3 [24]. We defined 0–5% of methylation as "unmethylated Foxp3".

### Murine mononuclear cell isolation and Treg and thymocyte studies

We housed C57BL/6 mice (The Jackson Laboratory, Bar Harbor, ME) in specific pathogen-free conditions and studied them using a protocol approved by the Institutional Animal Care and Use Committee of the Children's Hospital of Philadelphia. Spleen and lymph nodes were harvested, single cell suspensions prepared and magnetic beads (Miltenyi Biotec) used to isolate Treg cells (CD4+ CD25+), Teff cells (CD4+ CD25-), CD8+ T cells and APC (Thy1.2-). Generation of iTreg cells was performed as described [47]. Briefly, Teff cells were stimulated for 4 days with CD3ε/CD28 mAbs, in the presence of TGF-beta (3 ng/mL) and IL-2 (25 U/mL); we used the same conditions for generation of CD4+ and CD8+ iTreg cells. Murine Treg assays were performed as described [48]. Briefly, Teff cells were labeled with CFSE and stimulated for 72 h in the presence of irradiated APC (1∶1) plus CD3ε mAb (1 µg/mL), with or without Treg added at 1∶1-1∶8 (Treg: Teff) ratios. In some experiments, we used CellTrace (Invitrogen) to label Treg prior to these assays, so as to monitor Treg proliferation independently of dividing CFSE-labeled Teff cells. For thymocyte studies, thymi were harvested, single cell suspensions prepared, and cells were stimulated for 3 days with IL-2 (25 U/ml) with or without addition of CD3ε/CD28 mAbs.

### Flow Cytometry

We initially used anti-Helios mAbs generously gifted by Drs. Angela M. Thornton and Ethan M. Shevach of the National Institutes of Health, and later used the same Pacific Blue- and PE-labeled hamster anti-mouse/human Helios mAbs once they became commercially available (Biolegend). We purchased mAbs to human CD4 (APC), CD8 (FITC), CD25 (PE and APC), CTLA-4 (Pe-Cy5, PE) and Ki-67 (PerCP-Cy5.5) from BD Biosciences; CD4 (Pacific blue, PE, FITC, APC-eFluor 780), CD25 (APC-eFluor 780, Pe-Cy5, Pe-Cy7), CD39 (Pe-Cy7), CD45RA (PE-Cy7), CD45RO (PE), CD62L (Pe-Cy5, APC-eFluor 780), CD127 (FITC, PE, Alexa Fluor 647) and FOXP3 (PE, Alexa Fluor 647, Pacific blue and FITC, clones PCH101 and 236A/E7) from eBioscience; CD4 (PE-Texas Red) from Invitrogen; CD8 (PerCP/Cy5.5), CD31 (Pe-Cy7) and CD45RA (Pacific blue), from Biolegend; TNFRII/TNFRSF1B (CD120b, PE) from R&D and GARP (unconjugated and ATTO 488, Plato-1 clone) from Alexis Biochemicals. We purchased mAbs to murine CD4 (APC-eFluor 780, Pe-Cy5, Pacific blue, PE), CD8 (Pe-Cy7, PE, eFluor 605NC), CD25 (PE, PE-Cy5, PE-Cy7, APC-eFluor 780), CD44 (FITC), CD44RB (FITC), CD62L (APC-eFluor 780), Foxp3 (FJK-16a clone, Pe-Cy5, APC) and GARP (PE) from eBioscience. For all experiments, intranuclear staining for Foxp3 and Helios, and intracellular staining for CTLA-4 and Ki-67, were performed after surface staining; permeabilization was performed using the Foxp3 Fix/Perm Buffer kit (eBioscience). Cell fluorescence was measured using a Cyan flow cytometer (Dako) and data analyzed using Flowjo software (TreeStar). Most flow cytometry assays were performed with additional staining with LIVE/DEAD Fixable Aqua Dead Cell Stain Kit (Invitrogen) to improve the quality of gating live cells and decrease non-specific staining of dead cells.
